# Burnout and Professional Quality of Life Assessment in Portuguese Healthcare Professionals Working in Oncology and Palliative Care: A Cross-Sectional Study

**DOI:** 10.3390/healthcare13010026

**Published:** 2024-12-26

**Authors:** Florbela Gonçalves, Margarida Gaudêncio, Ivo Paiva, Miguel Castelo Branco, Joaquim Viana

**Affiliations:** 1 Faculty of Health Sciences, University of Beira Interior, 6201-001 Covilha, Portugal; mcbranco@fcsaude.ubi.pt; 2 Institute of Oncology Francisco Gentil Coimbra, 3000-075 Coimbra, Portugal; 4196@ipocoimbra.min-saude.pt; 3 Health Sciences Research Unit, Nursing, Nursing School of Coimbra, 3000-232 Coimbra, Portugal; ivopaiva@esenfc.pt; 4 Department of Anesthesiology, Coimbra Hospital and Universitary Center, 3004-561 Coimbra, Portugal; jsviana@fcsaude.ubi.pt

**Keywords:** burnout, compassion fatigue, emotional exhaustion, quality of life, work engagement

## Abstract

Introduction/Background: Burnout is a three-dimensional syndrome characterized by exhaustion that appears when the professional is constantly exposed to a stressful work environment, as well as depersonalization and lower personal accomplishment. Professional quality of life at work can be defined as the satisfaction degree that a person feels when being or going to their workplace. Objective: To evaluate burnout and professional quality of life in healthcare professionals working in oncology and palliative care. Material and methods: A cross-sectional, observational, and descriptive study was carried out in a convenience sample of 337 healthcare professionals from a Portuguese Oncology Hospital. The assessment tools were a sociodemographic questionnaire, the Maslach Burnout Inventory (MBI), and the Professional Quality of Life—version 5 (proQOL-5) scales. Statistical analysis was performed using the IBM SPSS^®^ Statistics program (significance level of 95% (*p* ≤ 0.05)). Results: The majority of professionals were female (84%), with a median age of 41 years. Most professionals work in oncological care services (76.8%), with an average of 40 h a week. There were no statistically significant differences in MBI and ProQOL dimensions between the two groups studied (professionals working in oncology vs. palliative care). In the sample and group of professionals working with palliative patients, it was observed that lower levels of compassion satisfaction were related to higher levels of emotional exhaustion (*p* < 0.001). On the other hand, higher levels of satisfaction correlated with a greater sense of personal accomplishment (*p* < 0.001); higher levels of secondary traumatic stress were related to a greater tendency towards emotional exhaustion (*p* < 0.001) and depersonalization (*p* = 0.031). Discussion and conclusions: Working in oncology and palliative care may predispose one to the onset of burnout related to contact with distress and suffering. With this study, the authors intend to demonstrate that both scales (MBI and ProQOL) are complementary in the analysis of the prevalence of burnout and professional quality of life, particularly in the context of oncology and palliative care. The risk of compassion fatigue and burnout highlights the need to develop coping strategies to minimize this risk and improve the quality of life and bonding of health professionals.

## 1. Introduction

Burnout is characterized by physical and psychological exhaustion related to chronic exposure to work-related stress [1,2,3,4]. The term was first introduced in the 1970s by Freudenberger and was later characterized by Maslach et al. as a three-dimensional syndrome: emotional exhaustion, depersonalization, and low personal accomplishment [1,2,3,4]. Burnout can occur in any type of profession [5].

Burnout syndrome is frequently observed in working environments where there is intense involvement with others, such as hospitals [6]. The burnout’s costs are growing fast, affecting 13–25% of the working population [7].

Several factors were found to mitigate/prevent burnout, such as co-worker social support, organizational support, positive team dynamics, effective communication, and recognition from a supervisor or manager [8].

Cancer is one of the main causes of mortality globally and is now considered to be a major public health problem worldwide [9]. Health professionals who deal with cancer patients, some of them in the palliative phase, face a highly demanding and stressful job given the suffering and proximity to death that many of these patients face [10].

The term compassion fatigue has emerged in the literature to describe exposure stress related to the suffering of others [11,12]. It is defined as a state of secondary trauma specific to professions in which there are helping relationships [11,12]. This phenomenon was first suggested by Charles Figley in 1995 after studying post-traumatic stress disorder in war veterans [11,12].

An even less studied subject in the literature is compassion satisfaction [13]. It is the opposite of burnout and compassion fatigue and means the satisfaction resulting from caring for others [13]. The prevalence of compassion fatigue varies according to the studies and where they are carried out, reaching values of 7.3% to 40% in Intensive Care Units [14] and around 60% in Palliative Care Units [15].

Quality of life at work can be defined by an employee’s level of satisfaction in their corporate environment, that is, the pleasure that a person feels when going to or being at their workplace [16].

In 2005, Stamm developed the Professional Quality of Life Scale (ProQOL), version 5, the most widely used (ProQOL-5) [17]. The ProQOL-5 was designed to assess the perception of quality of life in relation to the work [17]. This self-report instrument consists of three different subscales: burnout, secondary traumatic stress, and compassion satisfaction [15]. The ProQOL scale describes two fundamental aspects: compassion satisfaction, that is, the gratification that an employee feels when helping others, and compassion fatigue, which includes burnout and secondary traumatic stress [18].

Several measuring instruments have been developed to assess burnout and compassion fatigue. Bride et al. found six different scales that assessed the different domains of compassion fatigue [19]. However, only the ProQOL scale assesses all of these domains [17].

However, there are some investigations in which the psychometric validity of the ProQOL scale has been questioned, especially in the burnout dimension [18]. Heritage et al., when using the ProQOL scale on a sample of 1615 Australian nurses, created a revised and shorter version of only 21 items, which also assesses satisfaction and compassion fatigue [20]. Galiana et al. developed and validated a 9-item version of the ProQOL scale (the Short ProQOL) in a population of 1113 palliative care professionals with satisfactory results in monitoring occupational mental health [21].

Although the revised versions of ProQOL are promising, version 5 is still the one with the most evidence for measuring professional quality of life [22,23]. Studies carried out using ProQOL-5 have shown that a higher quality of professional life is associated with a lower prevalence of psychopathological conditions, such as depression and anxiety [22,23].

Several researchers have studied the correlation between quality of professional life, burnout, and other psychological aspects, such as depression and anxiety, in the field of oncology and palliative care [24,25].

However, no research has studied burnout (assessed by the Maslach Burnout Inventory—MBI) and professional quality of life (assessed by ProQOL-5) in healthcare professionals exposed to suffering, particularly in the oncological field. Health professionals in the area of oncology and palliative care are exposed to human suffering at different stages of the oncological disease since both areas are a continuum of care. It is thought that professionals linked to palliative care are more exposed to this suffering due to the fact that they come into contact with patients in more advanced stages of cancer. For this reason, the authors decided to develop a study to evaluate burnout and professional quality of life in healthcare professionals working in oncology and palliative care.

The specific objectives were to analyze the burnout and professional quality of life dimensions in healthcare professionals working with oncology patients (in different contexts of disease), particularly in palliative care. On the other hand, the authors tried to understand whether there was any correlation between the different dimensions of burnout and professional quality of life, measured by the MBI and ProQOL scale.

## 2. Material and Methods

### 2.1. Study Design

The authors presented a descriptive cross-sectional study among healthcare professionals working with oncology and palliative patients in Portugal.

### 2.2. Sample, Participants Recruitment Technique and Eligibility Criteria

The authors carried out a convenience sampling technique among healthcare professionals working in a Portuguese Oncological Hospital. Inclusion criteria encompassed professionals aged ≥ 18 years (working in this hospital) who consented to participate and comprehended the study’s objectives. Excluded were professionals under 18 years old, those unwilling to participate, and those with psychiatric disorders.

The authors suggested that professionals with psychiatric disorders mark the beginning of the questionnaire with a cross. These professionals are able to care for patients. The authors found in the literature that there is a significant level of discussion about the associations and distinctiveness of burnout with other mental health problems [26].

A 2018 systematic review indicated that the heterogeneity of published research does not allow a reliable examination of comorbidities, raising questions about whether it is possible to clearly distinguish burnout as an occupational syndrome from potentially underlying comorbidities [26].

For this reason, the authors decided to exclude the participants with mental health disorders in order to avoid some bias in the results.

### 2.3. Data Collection Tools

Data collection respected the guidelines outlined in the Helsinki Protocol [27] and the Oviedo Convention [28]. The study received approval from the ethics and management committee of the hospital (opinion n° T1 02/17).

The measurement tools were distributed individually, along with a letter explaining the study’s nature, objectives, and data confidentiality. Upon obtaining informed consent, data were collected using a structured evaluation protocol. This protocol included a sociodemographic and professional questionnaire with independent variables. As personal-related characteristics, the authors considered the following variables: gender, age, number of children, and marital status. On the other hand, as work-related characteristics, the authors considered these variables: a place of work (professionals who work or have worked in oncology departments vs. palliative unit), weekly workload, employment status, professional category, management status, years of experience, weekly workload, sleep duration, and night shifts. Additionally, the burnout and professional quality of life assessments were included, using the Maslach Burnout Inventory (MBI) and Professional Quality of Life—version 5 (proQOL-5) scales.

The authors conducted the assessments to ensure participant anonymity, recognizing the sensitive nature of the questions involved.

### 2.4. Measures Tools

The Maslach Burnout Inventory (MBI) is a self-reported burnout measure that includes three dimensions: emotional exhaustion (EE), depersonalization (DP), and personal accomplishment (PD), each assessed using various items [29]. The authors employed a validated language version (in Portugal) [29].

The emotional exhaustion dimension measures feelings of being emotionally overextended and exhausted by work [29]. On the other hand, depersonalization measures an unfeeling and impersonal response toward care treatment or instruction [29]. Personal accomplishment measures feelings of competence and successful achievement in work [29]. Scores for the 22 items range from 0 to 6, with specific ranges used to calculate scores for each dimension: emotional exhaustion (0–54), depersonalization (0–30), and personal accomplishment (0–48) [29]. MBI levels can be categorized as follows: emotional exhaustion (low: score ≤ 13; moderate: score 14–26; high: score ≥ 27), depersonalization (low: score ≤ 5; moderate: score 6–9; high: score ≥ 10), and personal accomplishment (low: score ≤ 33; moderate: score 34–39; high: score ≥ 40) [29]. The authors decided to present the burnout results according to these levels (categorical variables). Burnout is defined as high levels of emotional exhaustion and depersonalization combined with low levels of personal accomplishment [29].

The Professional Quality of Life Scale, version 5 (ProQOL-5), is a 30-item self-report measure composed of three subscales: compassion satisfaction (questions 3, 6, 12, 16, 18, 20, 22, 24, 27, 30), burnout (1, 4, 8, 10, 15, 17, 19, 21, 26, and 29), and secondary traumatic stress (STS) (questions 2, 5, 7, 9, 11, 13, 14, 23, 25, and 28) [17,30].

According to the definition of the PROQOL scale, compassion satisfaction is “about the pleasure you derive from being able to do your work well” [17,30]. On the other hand, burnout is considered an element of compassion fatigue [15,27]. “It is associated with feelings of hopelessness and difficulties in dealing with work or in doing your job effectively” [17,30]. At last, secondary traumatic stress is the second component of compassion fatigue [15,27]. “It is about your work-related, secondary exposure to extremely or traumatically stressful events” [17,30]. Participants were encouraged to indicate the score of each item in the previous 30 days on a 5-point Likert scale (from 1 = never to 5 = very often) [17,30]. The total score is the sum of the responses for each 10-item subscale [17,30]. The scores can be categorized as follows: STS (≤22 low; 23–41 moderate; ≥42 high); CS (≤22 low; 23–41 moderate; ≥42 high); and BO (≤22 low; 23–41 moderate; ≥42 high) [17].

### 2.5. Internal Consistency

Descriptive statistics were computed for each item within the scales to demonstrate the reliability of the mean values observed, considering that extreme cases can significantly influence these statistics.

Correlation coefficients between each pair of items within the same dimension were examined using Kendall’s Tau-b coefficient, as each item has up to five different response values. In terms of Cronbach’s alpha, commonly accepted criteria were followed: values above 0.80—desirable, above 0.70—recommended, and above 0.60—acceptable. Therefore, within the scope of this study, a Cronbach’s alpha result greater than 0.60 indicates satisfactory internal consistency.

Regarding the internal consistency of the items in each dimension of the MBI, Cronbach’s coefficients were 0.896 for emotional exhaustion, 0.663 for depersonalization, and 0.874 for personal accomplishment. As for ProQOL-5, Cronbach’s coefficients were 0.845 for compassion satisfaction, 0.749 for burnout, and 0.852 for secondary traumatic stress.

### 2.6. Statistical Analysis

Sample variables were characterized using the most appropriate descriptive statistics available. Data organization was performed using Microsoft Excel^®^ 2013 software.

Descriptive analysis was carried out by calculating absolute and relative frequencies (N and %) of categorical and qualitative variables. Quantitative variables were characterized by mean, quartiles, minimum, and maximum values. The categorical variables were analyzed considering the χ^2^ test (comparison test). For continuous variables, the means were analyzed using the *t*-test for independent samples (comparison test). In the case of qualitative variables, the Mann–Whitney test was used to compare the results.

Correlations between quantitative variables were performed using the Spearman correlation coefficient (non-normal distribution of data). Finally, the one-way ANOVA test was performed to better characterize MBI dimensions and their relationship with personal and work-related variables.

Statistical analysis was performed using the IBM SPSS^®^ Statistics program (version 25.0 for Windows^®^). The tests were performed at a significance level of 95% (*p* ≤ 0.05).

### 2.7. Ethical Consideration

Participation in the study was voluntary. Participants’ confidentiality and anonymity were guaranteed at all stages of the study, using exclusive code numbers and storing data on password-protected laptops with access only to researchers. Participants were not directly or indirectly involved in the design, implementation, reporting, or dissemination plans of the investigation.

## 3. Results

### 3.1. Participants

**Figure 1:**
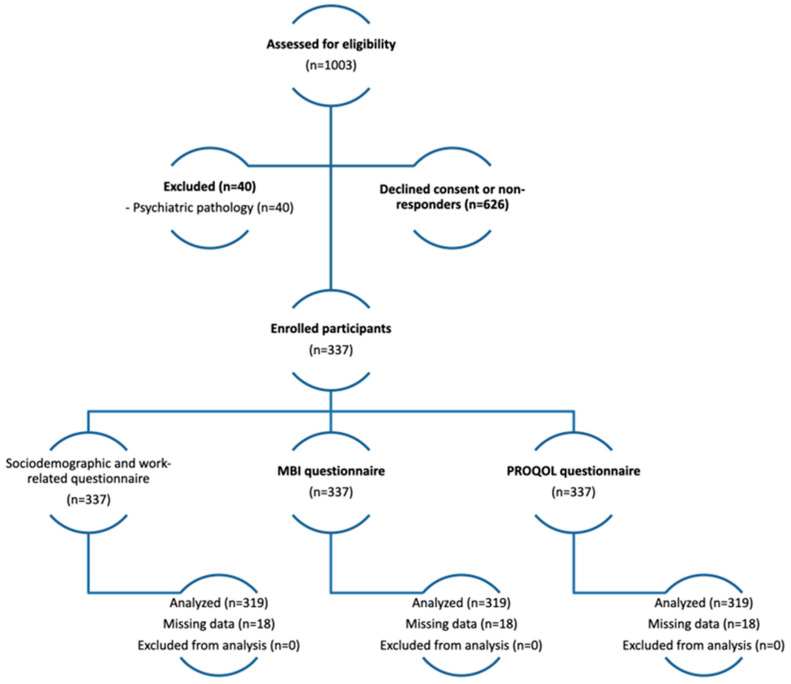
Participants’ flow diagram (sample selection process and follow-up).

The sample selection process and follow-up are described in Figure 1.

This study was conducted at a Portuguese tertiary hospital dedicated to cancer treatment, involving a total population of 1003 professionals. The authors recruited all professionals involved in direct care for cancer patients. Only 337 professionals were included since 40 presented psychopathology, and 626 declined consent or did not respond to the questionnaire. The response rate was 36%.

The authors would like to analyze the group of professionals who did not respond or decline consent, but we were not provided with the professional category of all members of the population (assessed for eligibility). Therefore, we only have the sociodemographic characterization of the professionals who actually participated in the study.

### 3.2. Personal and Work-Related Characteristics of the Sample (Sociodemographic Data)

In terms of personal characteristics, the majority of professionals were female (*n* = 269, 84%), with a median age of 41 years (from 20 to 69 years) (Table 1).

Most professionals have up to two children (*n* = 304, 95.2%) (Table 1). Regarding marital status, the majority of professionals were married (*n* = 204, 63.9%) or single (*n* = 82, 25.7%) (Table 1).

With regard to professional characteristics, most professionals work in services linked to oncological care (*n* = 245, 76.8%) (Table 2). These professionals work an average of 40 h a week (Table 2).

The sample consists of 38 doctors (11.9%), 149 nurses (46.7%), 68 operational assistants (21.3%), and 64 from other professional categories (social worker (*n* = 6), spiritual assistant (*n* = 1), pharmaceutics and diagnostic technicians (*n* = 51), secretary (*n* = 5), director (*n* = 1) (Table 2).

Most professionals do not work night shifts (*n* = 189, 59.2%) and have been working at this hospital for more than 10 years (*n* = 212, 66.5%). In relation to the employment link, 295 (92.5%) professionals have a contractual relationship (Table 2). Most professionals presented a sleep time between 6 and 8 h (*n* = 233, 73%) (Table 2).

### 3.3. Burnout Assessment Using the Maslach Burnout Inventory (MBI)

There were no statistically significant differences in MBI dimensions between the two groups studied (oncology services vs. palliative care) (Table 3).

In the emotional exhaustion dimension, medium and high values are presented in a third of professionals in each group, while approximately the same percentage is found in low levels (30%; *p* = 0.743) (Table 3).

About 20–25% of professionals presented depersonalization’s dimension with medium and high values, with around 50% of the sample showing low levels (*p* = 0.435).

As for personal accomplishment, there are no significant differences (*p* = 0.865), with identical values at the three levels in each of the groups (Table 3).

In the sample, 28 professionals presented criteria for burnout (8.8%).

### 3.4. Professional Quality of Life Assessment Using the Professional Quality of Life Scale (ProQOL)

Analysis of the ProQOL-5 scale dimensions reveals no significant differences between professionals in oncology and palliative care (*p* > 0.05) (Table 4 and Table 5).

Specifically, for compassion satisfaction, average levels are observed in approximately 75–76% of individuals in both groups, high levels in 4–6%, while low levels are reported by less than 6% (*p* = 0.794) (Table 5). Burnout is also comparable between the groups, with average levels observed in approximately 96% of professionals and low and high levels in less than 4% of the sample (*p* = 0.618) (Table 5). Finally, in terms of secondary traumatic stress, there are no significant differences (*p* = 0.625), with identical results (average and high levels observed in half of the sample and less than 1.2% in both groups) (Table 5).

### 3.5. Correlation Between the MBI Scale (Burnout) and ProQOL 5 (Professional Quality of Life)

Table 6 shows the correlation between the dimensions of the MBI and ProQOL in the sample and the group of professionals working in oncology vs. palliative care.

In the sample, it was observed that lower levels of compassion satisfaction are related to higher levels of emotional exhaustion (MBI dimension) (*p* < 0.001) (Table 6). Lower levels of burnout (according to ProQOL) are related to a greater tendency towards emotional exhaustion (*p* < 0.001) and depersonalization (*p* < 0.001), as well as a lower sense of personal accomplishment (*p* < 0.001) (Table 6). Higher levels of secondary traumatic stress are related to a greater tendency to emotional exhaustion (*p* < 0.001) and depersonalization (*p* < 0.001), as well as a lower sense of personal accomplishment (*p* < 0.001) (Table 6).

The correlations between MBI and ProQOL in the group of professionals working in oncology are not statistically significant, contrary to what happens in the population of professionals working in palliative care.

In the group of professionals working in palliative care, the results were similar to those observed in the sample. Lower levels of compassion satisfaction correlate with higher levels of emotional exhaustion (*p* < 0.001) and depersonalization (*p* < 0.001) (Table 6). Higher levels of satisfaction correlate with a greater sense of personal accomplishment (*p* < 0.001) (Table 6). Higher levels of burnout (according to ProQOL) are related to a greater tendency towards emotional exhaustion (*p* < 0.001) and depersonalization (*p* < 0.001), as well as a lower sense of personal accomplishment (*p* < 0.001) (Table 6). Higher levels of secondary traumatic stress are related to a greater tendency towards emotional exhaustion (*p* < 0.001) and depersonalization (*p* = 0.031) (Table 6). There was no statistically significant correlation between secondary traumatic stress and personal accomplishment (*p* = 0.142) (Table 6).

### 3.6. Determination of Some Potential Predictors of Burnout

In this chapter of the work, the authors decided to correlate the different MBI dimensions with personal and work-related characteristics of the total sample (professionals working in oncology and palliative care) (Table 7).

With regard to personal-related characteristics, the older the participants, the lower the level of emotional exhaustion (*p* < 0.001) and depersonalization (*p* < 0.05). Emotional exhaustion, depersonalization, and personal accomplishment are equally distributed between genders (Table 7).

On the other hand, the participants’ marital status and number of children present significant differences in terms of emotional exhaustion. Married participants perceive greater emotional exhaustion than single participants (Supplementary Table S1). Professionals who have two children perceive a greater level of emotional exhaustion than participants who do not have children (Supplementary Table S2).

With regard to work-related characteristics, there are no significant differences between the MBI dimensions and the following variables: professional category, years of work, employment link, management position, extra-work activities, and sleep hours per day.

So, this means that emotional exhaustion, depersonalization, and personal accomplishment are equally distributed among these professional characteristics.

Furthermore, with regard to weekly workload, there are no significant differences in terms of correlation (Table 7). However, when the authors carried out a more detailed analysis, they observed that professionals who work 40 h perceive greater emotional exhaustion than those who work 35 h (Supplementary Table S3). Professionals who work 40 h perceive greater depersonalization than those who work 35 h, and they perceive greater depersonalization than those who work 30 h (Supplementary Table S4). Finally, participants who work 22 h perceive greater personal accomplishment than those who work 37 h, and they perceive greater personal accomplishment than those who work 48 h (Supplementary Table S5).

## 4. Discussion

Burnout syndrome is frequently observed in working environments having intense involvement with others, such as oncology and palliative care [6].

According to the literature, these professionals are deeply affected by the suffering they witness and, over time, can begin to feel emotionally and psychologically exhausted, cynical, apathetic, depersonalized, and dissatisfied with their own work performance [8].

One of the study’s objectives was to compare the prevalence of burnout dimensions in the two groups studied (oncology vs. palliative care professionals).

In this investigation, none of the MBI burnout dimensions presented significant results. Both groups showed similar proportions for emotional exhaustion (medium and high values). Depersonalization was less common (20–25% of professionals) in both professionals. Additionally, there were no differences in personal accomplishment values.

Gómez-Urquiza et al.’s meta-analysis presented a prevalence estimation of 24% for emotional exhaustion (95% CI 16–34%), 30% for depersonalization (95% CI 18–44%), and 28% for low personal accomplishment, with a sample of 693 palliative care nurses [31].

Gonçalves et al. investigated the profile of burnout in palliative care professionals (physicians and nurses) during the COVID-19 pandemic using the Copenhagen Burnout Inventory scale [32,33]. Similar results were found in the work, personal, and patient dimensions among doctors (52%, 43%, and 21%) [32] and nurses (46%, 44%, and 22%) [33].

It is important not to forget that the COVID-19 pandemic represented a challenge for mental health on a global scale [34].

In the recent period, due to the COVID-19 pandemic, the world has experienced an unprecedented global public health crisis, with significant pressure on the healthcare system. In fact, the very high number of confirmed cases has had a huge impact on healthcare systems, which have seen the rationing or cessation of routine services, reorientation of clinical areas, redeployment of staff, shortages of personal protective equipment, and extensive responsibilities with medical resources and services pushed to maximum capacity due to unprecedented demands. Frontline healthcare professionals involved in the management and diagnosis of COVID-19 have been exposed to overwhelming pressure with consequent psychological stress. Since the COVID-19 pandemic, a great deal of evidence has been generated on burnout in healthcare professionals, leading to discussion on how to address it in some contexts [34].

So, this study found similar results in burnout dimensions compared to others in the literature. However, in the sample, the authors were able to verify that working exclusively with patients in palliative care does not represent an additional risk factor in relation to other professionals in oncology services. Indeed, in the sample, when the authors add the three dimensions of burnout, 8.8% of professionals experienced high levels of emotional exhaustion, high levels of depersonalization, and low levels of personal accomplishment.

Before continuing to analyze the results obtained, the authors would like to present a study carried out by a Portuguese team that found that working in palliative care was a protective factor for the development of burnout in nurses [35,36]. These professionals had lower emotional exhaustion values compared to their colleagues from other services like hematology and internal medicine [36]. Pereira et al. also found that palliative care professionals had a lower prevalence of burnout than intensive care professionals [35].

Another study’s objective was to evaluate the professional quality of life of healthcare professionals working with oncology and palliative patients.

In this research, the two groups (professionals working in oncology care vs. palliative care) do not present significant results regarding professional quality of life (measured by the three dimensions of the ProQOL scale). It was found that moderate levels were the most prevalent, being present in about 76% of compassion satisfaction, 96% for burnout, and about half for secondary traumatic stress in both groups (oncology vs. palliative care professionals).

In the literature, it was found that the prevalence of compassion satisfaction, burnout, and secondary traumatic stress was 22.89%, 62.79%, and 66.84%, respectively [37]. These results were described in a review and meta-analysis of fifteen studies (sample size—2509) [37].

Gerber et al. performed a study with professionals involved in pediatric palliative care [38]. These authors found low to moderate levels of burnout and secondary traumatic stress and moderate to high levels of compassion satisfaction [39].

Arimon-Pagès et al. investigated the emotional impact and compassion fatigue in oncology nurses (a multicenter study) [40]. It was found that of the 297 participants, 18.2% had low levels of compassion satisfaction, 20.2% had high levels of burnout, and 37.4% had high levels of secondary traumatic stress [40].

Frey et al. carried out a study on the nurses’ quality of professional life, but now, for those working in palliative care [38]. Around 48.4% of professionals had moderate–high levels of compassion satisfaction [38].

On the other hand, Kaur et al. reported high levels of compassion satisfaction (49.2%) in palliative healthcare professionals [41].

At last, the authors tried to correlate the three dimensions of the two scales (ProQOL and MBI) in the professionals working in oncology and palliative care and in the total sample. Although both scales measure the same concept, the authors sought to understand whether these scales are useful for assessing the prevalence of burnout and professional quality of life in the context of oncology and palliative care.

In the total sample and group of professionals working in palliative care, there was a significant relationship between the prevalence of the MBI dimensions and professional quality of life (ProQOL). The results of this analysis are in line with the authors’ expectations. Thus, when professionals are experiencing burnout (defined as high emotional exhaustion, cynicism, and low personal accomplishment), satisfaction with compassion (the pleasure of helping others at work) decreases.

However, in the group of professionals working in oncology care, the correlations between MBI and ProQOL are not statistically significant.

Based on this study, the prevalence of burnout measured by both scales (MBI and ProQOL) has uniform results, especially in the group of professionals who care for palliative patients.

With this analysis, the authors sought to demonstrate that both scales are complementary in the study of the prevalence of burnout and professional quality of life, particularly in the context of oncology and palliative care. Interestingly, it seems to us that using the scales together can improve the assessment of burnout and quality of professional life, particularly in groups of healthcare professionals exposed to more human suffering.

Exposure to traumatic events, such as dealing with patients in intense suffering, predisposes health professionals to become more exhausted and depersonalized. The lack of a statistically significant correlation between secondary traumatic stress and personal accomplishment may mean that these professionals already have effective coping mechanisms.

In this research, we intend to carry out an analysis of the reality of these professional groups. Both burnout and quality of professional life are influenced by individual and professional factors, which were not analyzed in this study, having already been the subject of a previous study.

In order to identify some potential predictors of burnout among professionals working in oncology and palliative care, the authors decided to correlate the MBI dimensions with personal and work-related characteristics.

With regard to personal-related characteristics, it was found that age, marital status, and participants’ number of children influenced the level of emotional exhaustion and depersonalization.

With regard to work and organizational-related characteristics, it was found that weekly workload influenced the different MBI dimensions.

These results are in line with data from the literature. Edú-Valsania et al. performed a review about burnout, where they addressed some factors related to it [42].

Workload, when excessive, requires physiological and psychological effort [42]. So, the working hours conditions (e.g., shift work, long working hours, or a large amount of overtime) that make it difficult to reconcile personal and professional life are an important trigger of burnout [42].

On the other hand, individual factors, such as personality traits and sociodemographic characteristics, could also predispose the development of burnout [42]. However, the results are not always so consistent [42].

In the literature, some strategies were described to help healthcare professionals prevent burnout and worse professional quality of life.

Development of personal skills in mindfulness, meditation, and coping may help fight against emotional exhaustion and burnout [8].

A supportive work environment is important, particularly to healthcare professionals, because positive interdisciplinary team dynamics help mitigate burnout [8].

On the other hand, healthcare organizations must take measures to create a culture of wellness [8]. Some measures should include limiting overtime, strengthening teams, offering stress management/stress inoculation workshops, reducing caseload size or diversity, developing equitable and worker/family-friendly policies, providing adequate payment, role modeling positive communication skills, effective conflict mitigation, responsiveness, and promotion of equity [8].

## 5. Strengths/Limitations

The authors identify several limitations in this study.

First, the cross-sectional design conditions the ability to examine changes in the variables over time. The research design does not allow causal inferences to be made. This kind of study allows the observation of variables at a single moment, which translates into a greater speed of realization, lower cost, lower losses, and the possibility of direct observation of the phenomena to be analyzed (avoiding the bias arising from memory or registration failures or inadequate or missing further information) and allows a wide variety of alternative methods, which can be used to statistically analyze the data.

So, a longitudinal study is necessary to comprehend the risk and degree of burnout and professional quality of life during a period of time. With a longitudinal perspective, it could measure burnout levels (and associated factors) over time.

Second, the use of a convenience sample could be considered a limitation.

Convenience sampling is based on ease of access and availability of participants. In this case, the authors chose to select a sample in a known workplace and in the context they intended to study in order to observe habits in a more accessible way. Therefore, the authors decided to choose this type of sampling due to its speed, simplicity, low cost, and ease of access to participants.

However, the use of a convenience sample may have introduced selection bias. The heterogeneous sample does not allow for differentiating the prevalence of burnout and the evaluation of the quality of professional life in the different professional groups. However, this study considered that all professionals are in a multidisciplinary team context.

Third, the social desirability bias is not excluded in this study due to the questionnaire’s self-reporting by the participants.

Furthermore, the authors recognize that excluding professionals with psychiatric pathology, despite the aim of avoiding bias in the results, poses a risk of them having hidden this fact when responding to the questionnaire.

Despite these limitations, the study has strengths as well. The use of validated instruments to measure both the risk of burnout and professional quality of life has enhanced the comparability of the study’s results. This investigation seeks to understand the usefulness of these two scales in the context of oncology and palliative care, particularly in the Portuguese healthcare professional population.

It is also important to highlight that this study is a pioneer in the sense that it seeks to evaluate and understand the usefulness of jointly assessing burnout risk and professional quality of life in healthcare workers exposed to human suffering. The MBI scale is universally validated for studying the prevalence of burnout. On the other hand, the ProQOL scale also assesses the burnout dimension, adding two other dimensions, also important, for determining the healthcare professionals’ quality of life.

## 6. Conclusions

Continuous and prolonged contact with pain and distress conditions has a high negative psychological impact on professionals in the field of oncology and palliative care, which means that they are more susceptible to burnout.

In our study, there were no significant differences between oncology and palliative professionals in terms of the prevalence of burnout and professional quality of life. However, it was observed that there is a correlation between the prevalence of burnout and the quality of professional life in this type of professional exposed to suffering, particularly in the palliative care context.

Compassion fatigue is a progressive and cumulative process, which begins as a discomfort generated by the feeling of compassion, progressing to stress and later to fatigue [43]. It is characterized by the association of low levels of compassion satisfaction, high levels of secondary traumatic stress, and burnout [43].

Compassion satisfaction is a personal trait that has been linked to resilience when working with chronically ill and suffering patients [43]. Professionals with high levels of compassion satisfaction deal better with negative emotions that can arise from empathetic involvement [43].

In the future, the authors consider it beneficial to expand this type of study to a larger population in order to determine more accurately the role of working quality of life and the prevalence of burnout in health professionals exposed to suffering. The risk of compassion fatigue and burnout highlights the need to develop coping strategies to minimize this risk and improve the quality of life and bonding of health professionals. It would be beneficial to study the impact of certain interventions, such as mindfulness or individual/team therapy, on the response to burnout and as a response to improving levels of professional satisfaction, particularly in this context.

## Figures and Tables

**Table 1:** Personal-related characteristics of the sample.

Variables		Working in Oncology Care * (*n* = 245) †	Working in Palliative Care (*n* = 74)	Total † (*n* = 337)
Age (Years), median [Q1; Q3], min–max		42 [35; 49], 20–69	40 [31; 43], 22–65	41 [34; 49], 20–69
Gender, *n* (%)	Female	205 (83.7)	63 (85.1)	268 (84)
	Male	40 (16.3)	11 (14.9)	51 (16)
Number of children, *n* (%)	0	68 (27.8)	31 (41.9)	99 (31)
	1	76 (31.0)	23 (31.1)	99 (31)
	2	89 (36.3)	17 (23.0)	106 (33.2)
	3	10 (4.1)	3 (4.1)	13 (4.1)
	4	1 (0.4)	0 (0)	1 (0.3)
	5	1 (0.4)	0 (0)	1 (0.3)
Marital status, *n* (%)	Single	60 (24.5)	22 (29.7)	82 (25.7)
	Divorced	24 (9.8)	5 (6.8)	29 (9.1)
	Window	3 (1.2)	1 (1.4)	4 (1.3)
	Married	158 (64.5)	46 (62.2)	204 (63.9)

† Missing—18. * Oncology, surgery, and radiotherapy services.

**Table 2:** Work-related characteristics of the sample.

Variables		Working in Oncology Care * (*n* = 245) †	Working in Palliative Care (*n* = 74)	Total (*n* = 337) †
Weekly workload (h), median [Q1; Q3], min–max		40 [35; 49], 28–49	40 [31; 43], 8–42	40 [35; 40], 8–49
Professional Category, *n* (%) †	Physician	30 (12.2)	8 (10.8)	38 (11.9)
	Nurse	108 (44.1)	41 (55.4)	149 (46.7)
	Operational assistant	52 (21.2)	16 (21.6)	68 (21.3)
	Other **	55 (22.4)	9 (12.2)	64 (20.1) **
Night work, *n* (%) ^1^	Yes	82 (34.0)	44 (59.5)	126 (39.5)
	No	159 (66)	30 (40.5)	189 (59.2)
Years of work, *n* (%) ^2^	≤3 years	24 (9.8)	20 (27)	44 (13.8)
	4–5 years	7 (2.9)	1 (1.4)	8 (2.5)
	6–10 years	41 (16.8)	13 (17.6)	54 (16.9)
	>10 years	172 (70.5)	40 (54.1)	212 (66.5)
Management position, *n* (%) ^3^	Yes	30 (12.5)	8 (11.3)	38 (11.9)
	No	210 (87.5)	63 (88.7)	273 (85.6)
Employment link, *n* (%) ^4^	Yes	229 (96.2)	66 (89.2)	295 (92.5)
	No	9 (3.8)	8 (10.8)	17 (5.3)
Extra-work activities, *n* (%) ^5^	Yes	94 (39.2)	34 (46.6)	128 (40.1)
	No	146 (60.8)	39 (53.4)	185 (58)
Sleep hours per day, *n* (%) ^1^	≤6 h	58 (24.0)	17 (23.3)	75 (23.5)
	>6 h–≤8 h	180 (74.4)	53 (72.6)	233 (73)
	>8 h	4 (1.7)	3 (4.1)	7 (2.2)

* Oncology, surgery, and radiotherapy services. † missing—18. ^1^ missing—22; ^2^ missing—19; ^3^ missing—26; ^4^ missing—25; ^5^ missing—24. ** social worker (*n* = 6), spiritual assistant (*n* = 1), pharmaceutics and diagnostic technicians (*n* = 51), secretary (*n* = 5), director (*n* = 1).

**Table 3:** MBI burnout levels (categorical variables) between the two groups (professionals working in oncology vs. palliative care).

Burnout MBI (Categorical)		Working in Oncology Care (*n* = 245) *,†	Working in Palliative Care (*n* = 74)	*p* **
Emotional exhaustion, *n* (%)	Low (≤13)	65 (29.1)	19 (28.4)	0.743
	Moderate (14–26)	79 (35.4)	27 (40.3)	
	High (≥27)	79 (35.4)	21 (31.3)	
	Total	223 ^1^	67 ^2^	
Depersonalization, *n* (%)	Low (≤5)	130 (59.6)	34 (50.7)	0.435
	Moderate (6–9)	46 (21.1)	17 (25.4)	
	High (≥10)	42 (19.3)	16 (23.9)	
	Total	218 ^3^	67 ^2^	
Personal accomplishment, *n* (%)	Low (≤33)	80 (36.9)	23 (34.3)	0.865
	Moderate (34–39)	64 (29.5)	22 (32.8)	
	High (≥40)	73 (33.6)	22 (32.8)	
	Total	217 ^4^	67 ^2^	
Burnout (High EE, High DP, and Low PA)—28 (8.8)				

* Missing—18; ** The comparison between the two groups was performed using the asymptotic chi-square test. ^1^—40 missing; ^2^—7 missing; ^3^—45 missing; ^4^—46 missing; †—oncology, surgery, and radiotherapy services. EE—emotional exhaustion; DP—depersonalization; PA—personal accomplishment.

**Table 4:** Professional quality of life of health professionals working in oncology and palliative care services (PROQOL—continuous variables).

PROQOL (Continuous Variables)	Working in Oncology Care (*n* = 245) *,†	Working in Palliative Care (*n* = 74)	*p*-Value **
PROQOL (compassion satisfaction), median [Q1; Q3], min–max	36 [33; 40], 0–50	37.5 [33; 41], 0–50	0.552
PROQOL (burnout), median [Q1; Q3], min–max	31 [28; 34], 0–45	31 [28; 34], 0–43	0.993
PROQOL (secondary traumatic stress), median [Q1; Q3], min–max	23 [18; 28], 0–48	23 [19.75; 27.25], 0–35	0.701
PROQOL (total score), median [Q1; Q3], min–max	90 [83.5; 98], 0–127	92 [86; 98], 0–118	0.664

* Missing—18; †—oncology, surgery, and radiotherapy services. ** *t*-test for independent samples.

**Table 5:** Professional quality of life of health professionals working in oncology and palliative care services (PROQOL—categorical variables).

PROQOL (Categorical Variables)		Working in Oncology Care (*n* = 245) *,†	Working in Palliative Care (*n* = 74)	*p*-Value **
PROQOL (compassion satisfaction), *n* (%)	Low (≤22)	14 (5.7)	3 (4.0)	0.794
	Moderate (23–41)	187 (76.3)	56 (75.6)	
	High (≥42)	44 (17.9)	15 (20.2)	
PROQOL (burnout), *n* (%)	Low (≤22)	9 (3.6)	2 (2.7)	0.618
	Moderate (23–41)	235 (95.9)	71 (95.9)	
	High (≥42)	1 (0.4)	1 (1.3)	
PROQOL (secondary traumatic stress), *n* (%)	Low (≤22)	117 (47.7)	35 (47.2)	0.625
	Moderate (23–41)	125 (51.0)	39 (52.7)	
	High (≥42)	3 (1.2)	0 (0.0)	

* Missing—18; †—oncology, surgery, and radiotherapy services.** Mann–Whitney test.

**Table 6:** Spearman correlation between ProQOL and MBI scales in the professionals working in oncology and palliative care and in the total sample—r (*p*-value).

MBI Dimensions		MBI EE			MBI DP			MBI PA		
Professionals		Work in Oncology	Work in Palliative	Total	Work in Oncology	Work in Palliative	Total	Work in Oncology	Work in Palliative	Total
ProQOL dimensions	PROQOL—CS	−0.131 (0.087)	−0.452 (<0.001)	−0.501 (<0.001)	−0.134 (0.082)	−0.403 (0.001)	−0.498 (0.001)	0.054 (0.485)	0.543 (0.001)	0.546 (<0.001)
	PROQOL—B	−0.039 (0.602)	0.658 (<0.001)	0.690 (<0.001)	0.036 (0.635)	0.504 (0.001)	0.536 (<0.001)	0.036 (0.636)	−0.587 (0.001)	−0.508 (<0.001)
	PROQOL—STS	−0.012 (0.875)	0.499 (<0.001)	0.449 (<0.001)	0.054 (0.484)	0.282 (0.031)	0.337 (<0.001)	0.007 (0.927)	−0.194 (0.142)	−0.274 (<0.001)

EE—emotional exhaustion; DP—depersonalization; PA—personal accomplishment; CS—compassion satisfaction; B—burnout; STS—secondary traumatic stress.

**Table 7:** Spearman correlation between burnout (MBI) with personal and work-related characteristics (in total sample)—r (*p*-value).

Personal and Work-Characteristics		Age	Gender	Marital Status	Number of Children	Weekly Workload	Professional Category	Years of Work	Night Work	Employment Link	Management Position	Extra-Work Activities	Sleep Hours Per Day
MBI burnout dimensions	MBI EE	−0.209 (<0.001)	−0.015 (0.807)	−0.129 (0.036)	−0.160 (0.009)	0.092 (0.135)	−0.026 (0.679)	−0.035 (0.569)	−0.062 (0.318)	−0.049 (0.434)	0.110 (0.078)	−0.035 (0.575)	−0.011 (0.861)
	MBI DP	−0.159 (0.011)	−0.003 (0.968)	0.002 (0.977)	−0.070 (0.265)	0.036 (0.561)	−0.006 (0.923)	−0.080 (0.202)	−0.030 (0.638)	−0.044 (0.487)	−0.037 (0.559)	−0.004 (0.953)	−0.105 (0.094)
	MBI PA	0.116 (0.063)	−0.073 (0.246)	0.000 (0.998)	0.019 (0.757)	−0.027 (0.667)	−0.048 (0.445)	0.004 (0.947)	0.043 (0.499)	0.008 (0.895)	0.097 (0.124)	0.028 (0.662)	0.021 (0.734)

EE—emotional exhaustion; DP—depersonalization; PA—personal accomplishment.

## Data Availability

The datasets generated and analyzed during the current study are available from the corresponding author upon reasonable request.

## Supplementary Materials

The following supporting information can be downloaded at: https://www.mdpi.com/article/10.3390/healthcare13010026/s1, Table 1: Relationship between MBI dimensions and personal-characteristics (marital status); Table 2: Relationship between MBI dimensions and personal-related characteristics (number of children); Table 3: Relationship between MBI Emotional exhaustion dimension and work—related characteristics (weekly workload); Table 4: Relationship between MBI Depersonalization and work—related characteristics (weekly workload); Table 5: Relationship between MBI Personal Accomplishment and work-related characteristics (weekly workload).
