# Supplementary material for: Burnout and Professional Quality of Life Assessment in Portuguese Healthcare Professionals Working in Oncology and Palliative Care: A Cross-Sectional Study
Source: Healthcare (Basel). 2024 Dec 26;13(1):26. doi: 10.3390/healthcare13010026 (PMC11719651; doi:10.3390/healthcare13010026)
Supplement: Supplementary file 1 [file healthcare-13-00026-s001.zip › healthcare-3337926-SI.pdf]

SUPPLEMENTARY MATERIAL

Table S1. Relationship between MBI dimensions and personal - related characteristics (marital status)

Pairwise comparisons (One-way Anova)

Sample 1-Sample 2	*Means differences*	*Standard Error*	*p*
Divorced-Widow	-0.399	41.636	0.992
Divorced - Married	-0.911	17.615	0.959
Divorced - Single	27.282	19.270	0.157
Widow - Married	-0.512	38.589	0.989
Widow - Single	26.883	39.372	0.495
Married - Single	26.371	11.263	0.019

Table S2. Relationship between MBI dimensions and personal-related characteristics (number of children)

Pairwise comparisons (One-way Anova)

**Sample 1-Sample 2**	*Means differences*	*Standard Error*	*p*
4-2	17.247	76.757	0.822
4-5	-37.000	107.932	0.732
4-1	37.134	76.762	0.629
4-3	43.409	79.713	0.586
4-0	51.686	76.807	0.501
2-5	-19.753	76.757	0.797
2-1	19.887	11.605	0.087
2-3	-26.162	24.423	0.284
2-0	34.439	11.901	0.004
5-1	0.134	76.762	0.999
5-3	6.409	79.713	0.936
5-0	14.686	76.807	0.848
1-3	-6.275	24.439	0.797
1-0	14.552	11.933	0.223
3-0	8.277	24.580	0.736

Table S3. **Relationship between MBI Emotional exhaustion dimension and work-related characteristics (weekly workload)**

Pairwise comparisons (One-way Anova)

Sample 1-Sample 2	*Means differences*	*Standard Error*	*p*
22-48	-27.500	107.524	0.798
22-37	-53.000	107.524	0.622
22-8	105.500	107.524	0.327
22-35	-107.161	76.642	0.162
22-30	-130.635	77.479	0.092
22-40	-130.953	76.268	0.086
22-28	-142.167	87.793	0.105
22-31	-157.250	93.119	0.091
22-42	-159.167	82.123	0.053
48-37	25.500	107.524	0.813
48-8	78.000	107.524	0.468
48-35	79.661	76.642	0.299
48-30	103.135	77.479	0.183
48-40	103.453	76.268	0.175
48-28	114.667	87.793	0.192
48-31	129.750	93.119	0.164
48-42	131.667	82.123	0.109
37-8	52.500	107.524	0.625
37-35	54.161	76.642	0.480
37-30	77.635	77.479	0.316
37-40	-77.953	76.268	0.307
37-28	89.167	87.793	0.310
37-31	104.250	93.119	0.263
37-42	-106.167	82.123	0.196
8-35	-1.661	76.642	0.983
8-30	-25.135	77.479	0.746
8-40	-25.453	76.268	0.739
8-28	-36.667	87.793	0.676
8-31	-51.750	93.119	0.578
8-42	-53.667	82.123	0.513
35-30	23.473	17.764	0.186
35-40	-23.792	11.374	0.036
35-28	35.005	44.946	0.436
35-31	50.089	54.622	0.359
35-42	-52.005	32.507	0.110

30-40	-0.319	16.077	0.984
30-28	11.532	46.360	0.804
30-31	-26.615	55.792	0.633
30-42	-28.532	34.435	0.407
40-28	11.214	44.306	0.800
40-31	26.297	54.097	0.627
40-42	-28.214	31.616	0.372
28-31	-15.083	69.407	0.828
28-42	-17.000	53.762	0.752
31-42	-1.917	62.079	0.975

Table S 4. Relationship between MBI Depersonalization and work- related characteristics (weekly workload)

Pairwise comparisons (One-way Anova)

**Sample 1-Sample 2**	***Means differences***	***Standard Error***	***p***
48-22	73.500	104.778	0.483
48-28	86.667	85.551	0.311
48-35	88.367	74.704	0.237
48-40	115.253	74.326	0.121
48-42	126.417	80.026	0.114
48-30	131.885	75.501	0.081
48-31	170.250	90.741	0.061
48-8	186.500	104.778	0.075
48-37	197.500	104.778	0.059
22-28	-13.167	85.551	0.878
22-35	-14.867	74.704	0.842
22-40	-41.753	74.326	0.574
22-42	-52.917	80.026	0.508
22-30	-58.385	75.501	0.439
22-31	-96.750	90.741	0.286
22-8	113.000	104.778	0.281
22-37	-124.000	104.778	0.237
28-35	-1.700	43.832	0.969
28-40	-28.587	43.185	0.508
28-42	-39.750	52.389	0.448
28-30	-45.218	45.176	0.317

28-31	-83.583	67.634	0.217
28-8	99.833	85.551	0.243
28-37	-110.833	85.551	0.195
35-40	-26.887	11.255	0.017
35-42	-38.050	31.723	0.230
35-30	43.518	17.396	0.012
35-31	81.883	53.255	0.124
35-8	98.133	74.704	0.189
35-37	-109.133	74.704	0.144
40-42	-11.163	30.823	0.717
40-30	16.631	15.694	0.289
40-31	54.997	52.724	0.297
40-8	71.247	74.326	0.338
40-37	82.247	74.326	0.268
42-30	5.468	33.556	0.871
42-31	43.833	60.494	0.469
42-8	60.083	80.026	0.453
42-37	71.083	80.026	0.374
30-31	-38.365	54.367	0.480
30-8	54.615	75.501	0.469
30-37	-65.615	75.501	0.385
31-8	16.250	90.741	0.858
31-37	-27.250	90.741	0.764
8-37	-11.000	104.778	0.916

Table S 5. Relationship between MBI Personal Accomplishment and work-related characteristics (weekly workload)

Pairwise comparisons (One-way Anova)

Sample 1-Sample 2	*Means differences*	*Standard Error*	*p*
37-31	78.250	90.595	0.388
37-8	87.500	104.611	0.403
37-30	117.000	75.436	0.121
37-40	-117.696	74.208	0.113
37-35	133.517	74.585	0.073
37-42	-148.500	79.898	0.063
37-28	151.167	85.414	0.077

37-22	231.000	104.611	0.027
37-48	-244.000	104.611	0.020
31-8	9.250	90.595	0.919
31-30	38.750	54.357	0.476
31-40	-39.446	52.640	0.454
31-35	-55.267	53.170	0.299
31-42	-70.250	60.397	0.245
31-28	72.917	67.526	0.280
31-22	152.750	90.595	0.092
31-48	-165.750	90.595	0.067
8-30	-29.500	75.436	0.696
8-40	-30.196	74.208	0.684
8-35	-46.017	74.585	0.537
8-42	-61.000	79.898	0.445
8-28	-63.667	85.414	0.456
8-22	-143.500	104.611	0.170
8-48	-156.500	104.611	0.135
30-40	-0.696	15.936	0.965
30-35	-16.517	17.609	0.348
30-42	-31.500	33.628	0.349
30-28	34.167	45.197	0.450
30-22	114.000	75.436	0.131
30-48	-127.000	75.436	0.092
40-35	15.821	11.237	0.159
40-42	-30.804	30.774	0.317
40-28	33.471	43.116	0.438
40-22	113.304	74.208	0.127
40-48	-126.304	74.208	0.089
35-42	-14.983	31.672	0.636
35-28	17.650	43.762	0.687
35-22	97.483	74.585	0.191
35-48	-110.483	74.585	0.139
42-28	2.667	52.305	0.959
42-22	82.500	79.898	0.302
42-48	-95.500	79.898	0.232
28-22	79.833	85.414	0.350
28-48	-92.833	85.414	0.277
22-48	-13.000	104.611	0.901
